# The successional trajectory of bacterial and fungal communities in soil are fabricated by yaks’ excrement contamination in plateau, China

**DOI:** 10.3389/fmicb.2022.1016852

**Published:** 2022-11-17

**Authors:** Zhenda Shang, Yaping Wang, Miao An, Xiushuang Chen, Muhammad Fakhar-e-Alam Kulyar, Zhankun Tan, Suozhu Liu, Kun Li

**Affiliations:** ^1^College of Animal Science, Tibet Agricultural & Animal Husbandry University, Nyingchi, China; ^2^College of Veterinary Medicine, Huazhong Agricultural University, Wuhan, China; ^3^Institute of Traditional Chinese Veterinary Medicine, College of Veterinary Medicine, Nanjing Agricultural University, Nanjing, China; ^4^MOE Joint International Research Laboratory of Animal Health and Food Safety, College of Veterinary Medicine, Nanjing Agricultural University, Nanjing, China

**Keywords:** excrement contamination, plateau, soil microbiota, bacterial and fungal communities, yaks

## Abstract

The soil microbiome is crucial in determining contemporary realistic conditions for future terrestrial ecological and evolutionary development. However, the precise mechanism between the fecal deposition in livestock grazing and changes in the soil microbiome remains unknown. This is the first in-depth study of bacterial and fungal taxonomic changes of excrement contaminated soils in the plateau (>3,500 m). This suggests the functional shifts towards a harmful-dominated soil microbiome. According to our findings, excrement contamination significantly reduced the soil bacterial and fungal diversity and richness. Furthermore, a continuous decrease in the relative abundance of microorganisms was associated with nutrient cycling, soil pollution purification, and root-soil stability with the increasing degree of excrement contamination. In comparison, soil pathogens were found to have the opposite trend in the scenario, further deteriorating normal soil function and system resilience. Such colonization and succession of the microbiome might provide an important potential theoretical instruction for microbiome-based soil health protection measures in the plateau of China.

## Introduction

Soil harbors are home to various microorganisms, including bacteria and fungi, referred to as the soil microbiome (Geisen et al., 2015). Soil microbiome is a dynamic and complex ecosystem in the rhizosphere and/or in the bulk soil, which regulates primary production, organic-matter degradation and carbon sequestration in soil, etc., (Bardgett and van der Putten, 2014; Ruiz et al., 2020). The interaction relationships between below-ground and aboveground microorganisms influence the composition, production, succession formation, and dynamic changes in soil communities (Kardol and Wardle, 2010). Both deterministic and stochastic processes may influence community assembly processes in soil ecosystems. The former involves niche-based mechanisms, including natural selection (e.g., competition, inhibition, predation, and mutualisms), while the latter reflect random shifts in the relative abundances of microorganisms (Chase, 2010; Vellend, 2010; Rosindell et al., 2011). In turn, the activities and interactions of soil microorganisms have a major impact on life-sustaining processes such as soil productivity and nutrient cycling (Cardinale, 2012). The soil microbiome composition, e.g., bacteria and fungi, can be influenced by the competitive relationships arising from resource sharing or coexisting interactions (de Boer et al., 2005; Mille-Lindblom et al., 2006). However, our knowledge about this hidden biodiversity is less established than aboveground community dynamics (Bardgett and van der Putten, 2014). This inadequate understanding hampers us from anticipating the subsequent outcomes of livestock grazing, which induces a soil microbial diversity shift (Wardle, 2002).

Livestock production is indispensable to agriculture throughout the globe, especially in highland areas of China. The Qinghai-Tibet Plateau (>3,000 m) has extremely harsh living conditions, such as low temperature, hypoxia, and intense ultraviolet rays (Li et al., 2019). The local nomads carried on the ancient civilization, whose primary source of income was grazing yaks, a distinctive plateau species (Wang et al., 2021). Previous studies reported that the microbiome composition is influenced by environmental conditions (Li and Zhao, 2015). Another long-standing issue in micro-ecology is the intensity of grazing that poses a significant challenge and pressure on land. For example, animal movement and continuous grazing disrupted soil aggregates, resulting in a negative soil stability index associated with the variation in soil structure and/or soil porosity (Imhoff et al., 2000; Di et al., 2001). Aside from soil compaction, excremental contamination has reduced soil microbial diversity (Bardgett and van der Putten, 2014; Schutzius et al., 2019). Pathogenic bacteria (even anthropozoonosis) are carried in animal excrement that enters surface water or soil after surface runoff and percolation/infiltration after precipitation events, causing significant harm to human health (Harwood et al., 2014; Guo et al., 2019). Several methods have been developed to accomplish soil protection, one of which is to enhance soil immunity, i.e., the ability of soil to defend against invading pathogens (Raaijmakers and Mazzola, 2016). It has been proved that soil functional microorganisms’ activities determine soil immunity (Wang and Li, 2019). Here, characterizing the soil microbiome colonization and succession based on grazing-dependent impacts may be important if adopted a microbiome-based strategy to improve soil resilience and immunity.

Currently, studies related to soil microbial biodiversity are entering a new era. More and more scientists are becoming aware of the critical role of soil biodiversity in providing important ecological services to human society. They are using the latest advanced tools to explore the micro-biodiversity of soil (Larigauderie et al., 2012). Here we explore the variation in soil microbial community, including bacteria and fungi, caused by animal excrement contamination in plateau using 16S ribosomal DNA (16S rDNA) and Internal Transcribed Spacer (ITS) high-throughput sequencing techniques. Exploring interactions between excrement contamination and the soil microbial community may lead to novel ways to manage soil-borne diseases in the plateau.

## Materials and methods

### Soil sampling and processing

During May 2021, soil samples were collected at a place in Aba Autonomous Prefecture (31°51′-33°33’ N, 101°51′-103°22′ E, > 3,500 m). Furthermore, yaks in the sampling sites were allowed to graze freely without any treatment for their excrement. According to the natural habit of the yaks, all animals were grazed freely in the highland pasture (defined as NM, 12,000 m^2^ per yak) from 06:00 h and 18:00 h, and the rest of the day was in the housed area (defined as NJ, 3 m^2^ per yak). Total 18 soil cores (0–15 cm depths and 2.5 cm diameter) were collected randomly from the three plots by using sterile aluminum core tubes, no-grazing soil (NF), grazing effect soil (NM) and yaks’ housed area soil (NJ), with intact cores being delivered to the laboratory, homogenized and sieved (2.0 mm mesh) to remove plant debris and flash-subsequently frozen in −80°C for further analysis.

### DNA extraction

Through high-throughput sequencing, 16S rDNA and ITS genes characterized bacterial and fungal diversity. Following the instruction procedure, the genomic DNA (gDNA) was extracted per soil sample using FastDNA Spin Kit (MP Biochemicals, United States). Afterward, the purity of extracted DNA was done using a Universal DNA Purification Kit (TIANGEN Biotech Co., Ltd., Beijing, China). The quality and integrity of gDNA were evaluated by 1% agarose gel electrophoresis and UV–Vis spectrophotometer (Thermo Fisher Scientific, Nanodrop™, Waltham, United States).

### 16S rDNA and its genes amplification and sequencing

The specific primers based on the V3-V4 region for bacteria of the 16S rDNA gene and ITS region for fungi of the 18S rRNA gene were synthesized for PCR amplification (F: 5’-ACTCCTACGGGAGGCAGCA-3′ and R: 5’-GGACTACHVGGGTWTCTAAT-3′) and (F: 5’-NNNNNNNNGCATCGATGAAGAACGCAGC-3′ and 5’-TCCTCCGCTTATTGATATGC-3′), respectively (Hu et al., 2016). The melting temperature of bacterial was 55°C and PCR cycles were 35; whereas the melting temperature of fungal was 58°C for 50 s, and PCR cycles were 35. After cleaning and normalizing, subsequent quality control of PCR amplicons was executed using a High Sensitive DNA Kit (Agilent Technologies, United States). The sequencing library was prepared according to the standard process provided by Illumina (Illumina, Inc., SanDiego, California, United States). The qualified library was validated in the following rules: library with one peak and no linker was selected; using Library Quantification Kit (KAPA Biosystems, United Kingdom) to quantify the libraries and the concentrations were kept above 2 nM. Finally, the qualified library was sequenced paired ends on the Illumina Novaseq platform (Illumina, Inc., SanDiego, California, United States) to define the phylogenetic types.

In order to acquire more accurate and reliable sequencing results (effective reads), the raw data from high-throughput sequencing were filtered to eliminate the low quality by the following pre-procedures: (1) The raw reads were filtered using Trimmomatic software (v0.33, Germany), subsequent the primer sequences were identified and removed by using Cutadapt software (v1.9.1, Germany) to acquire clean reads, (2) Clean paired-end sequences were overlapped and merged to tags using Usearch software (v1.0, United States), (3) those reads contaminated by chimeric sequences were identified and removed with the UCHIME (v4.2, United States) tool to acquire the final effective reads. Subsequently, Quality control and taxonomically classified operational taxonomic unit (OTU, with 97% sequence similarity) mapping to the SILVA (for bacteria), and UNITE (for fungi) databases were performed using the QIIME platform (v1.8.0, United States).

### Bioinformatics and statistical analysis

Venn diagram, drawn using R software (v3.0.3, Austria), visually showed the counts of unique and common OTUs among groups. The Rarefaction curve, Shannon index, Rank abundance curve, and Coverage index were set for assessing high-throughput sequencing data volume, depth, richness, and uniformity of the species contained in the sample. The Quantitative Insights into Microbial Ecology (v1.8.0, QIIME, United States) was used for constructing a genus-level phylogenetic tree and subsequently drawn by Python software. ACE index, Chao1 index, Shannon index, and Simpson index, which reflect alpha diversity, were evaluated by QIIME software (v1.8.0, United States). The similarities and clustering between individuals or groups were visually exhibited using Principal Coordinates Analysis (PCoA) and Unweighted Pair-group Method with Arithmetic Mean (UPGMA). Metastats software was used to perform T-test for finding the differentially abundant taxa (phylum, class, and genus) among groups. Student’s t-test was used to compare the alpha diversities among groups and perform calculations in SPSS software (v17.0, United States). The values were expressed as the mean ± SD, and the value of *p* (corrected) less than 0.05 indicated statistical significance.

## Results

### 16S rDNA and its high-throughput sequence data analysis

**Figure 1:**
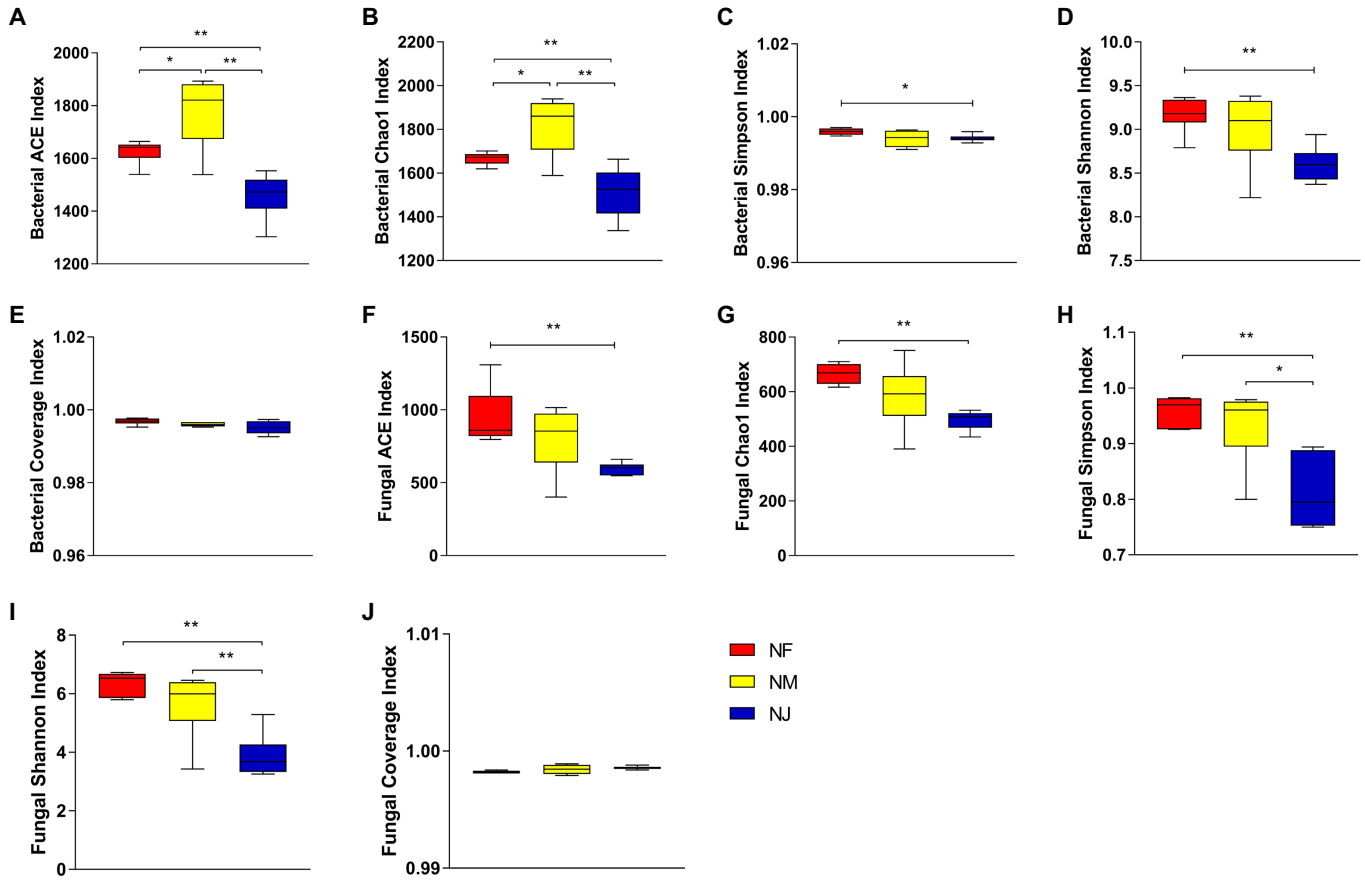
Evaluation of alpha diversity indices. **(A, C–F)** Represented soil bacterial, Coverage indices, ACE, Chao1, and Simpson, Shannon, respectively. **(B, G–H)** Represented fungal, Coverage indices, ACE, Chao1, Simpson, and Shannon, respectively. “*” represented that “*p* < 0.05”; “**” represented that “*p* < 0.01.”

A total of 1,318,076 and 1,412,260 high-quality bacterial and fungal reads were obtained after quality control, which was grouped into 2,065 and 1,139 OTUs. The average number of effective reads generated per sample was 73,226 and 78,459 from V4 and ITS regions, respectively (Supplementary Tables S1, S2). Multi-indicators, Rarefaction curve, Shannon index, and Species accumulation curve per sample tended to be flat and extended to the right end of the x-axis, indicating that almost all species were annotated in bacteria and fungi populations (Supplementary Figure S1). Furthermore, the estimated coverage rate (Coverage) values were greater than 99%, indicating that the sequencing depth sufficiently represented the diversity of bacterial and fungal communities (Figures 1A,B).

**Figure 2:**
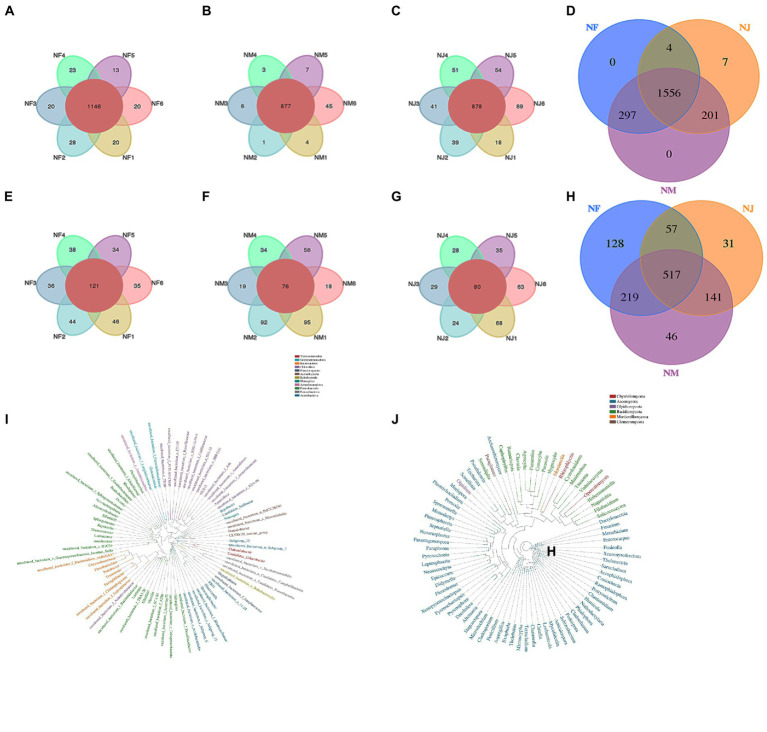
Analysis of Venn diagrams. **(A, C–E)** Venn diagrams for bacterial OTUs compositions in different groups. **(B, F–H)** Venn diagrams for fungal OTUs compositions in different groups.

**Figure 3:**
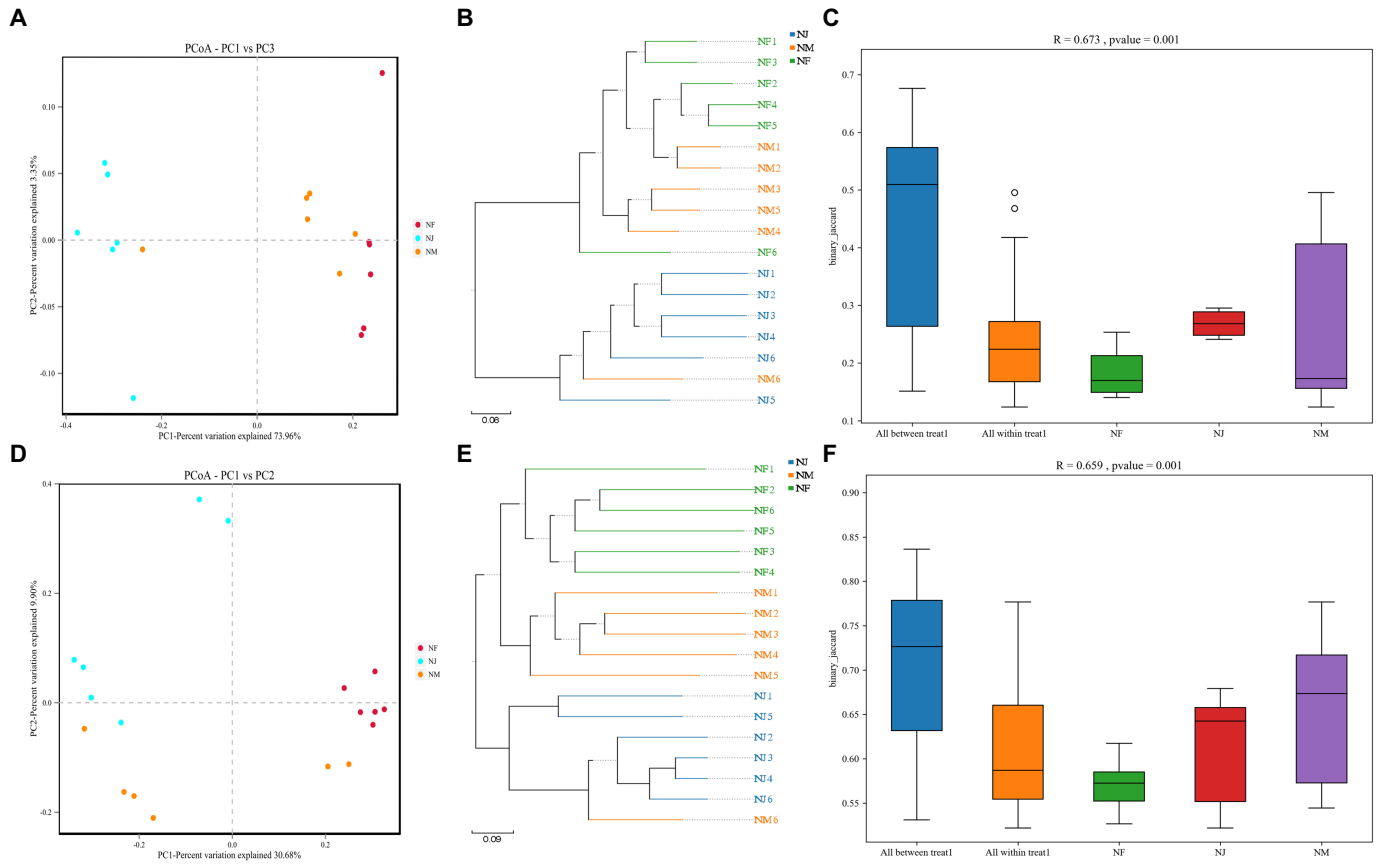
Similarity analysis of soil microbial structures in all groups. **(A–C)** represented the bacterial similarity between groups or individuals using PCoA, UPGMA, and Anosim box Figures. **(D–F)** represented the fungal similarity between groups or individuals using PCoA, UPGMA, and Anosim box Figures.

Among detected 3,204 OTUs clustered at 97% sequence similarity, 1,556 bacterial and 517 fungal core OTUs were identified in all soil samples, accounting for approximately 48.6 and 16.1% of the total OTUs (Figures 2A,B). As expected, we detected a far higher number of core OTUs than unique OTUs per group, indicating that the population differences between groups were higher than those within groups (Figures 2C–E, 2F–H). Using the Python program, the representative sequences of the genus classification level were subjected to numerous sequence alignments to build a phylogenetic tree and image based on the OTU classification (Supplementary Figures 2A,B). The results of Anosim (analysis of similarities) also support these speculations (Figures 3C,F).

### Changes in the diversity of bacterial and fungal communities under the influence of yaks’ excrement contamination

The relationship between soil bacterial and fungal alpha diversity and the yaks’ excrement contamination was explored in the three habitats’ soil layers. The ACE indices and Chao1 indices (reflected species richness), Shannon indices and Simpson indices (species diversity) of these three different soil communities were significantly different. Our results showed that the bacterial and fungal alpha diversity in NJ was lower than other soil properties. Specifically, the average bacterial ACE, Chao1, Shannon, and Simpson indices were 1626.326, 1666.811, 9.170, 0.996 in NF, 1778.276, 1818.304, 9.007, 0.994 in NM, whereas 1458.801, 1512.533, 8.602, 0.994 in NJ (Figures 1C–F). Results indicated that the soil bacterial microbiome in NF had the highest species diversity (*p* < 0.05 or *p* < 0.01). Unlike bacteria, NF soil had the highest fungal species richness and diversity among the three groups, followed by NM, while soil microbial alpha diversity in NJ comes last (Figures 1G–H).

The PCoA plots and UPGMA tree revealed the patterns of bacterial and fungal microbiota β diversity. Unsupervised clustering using PCoA based on UniFrac distance matrices suggested the variations in our dataset due to excrement contamination, with the bacterial and the fungal microbiota clustering independently from different groups (Figures 3A,D). Soil microbiome was sequestered into three branches based on shifts in evolutionary information per sample, indicating a difference in soil microbiome induced by excrement contamination (Figures 3B,E).

### Significant alterations bacterial-microbiota composition and dominant species under the influence of yaks’ excrement contamination

**Figure 4:**
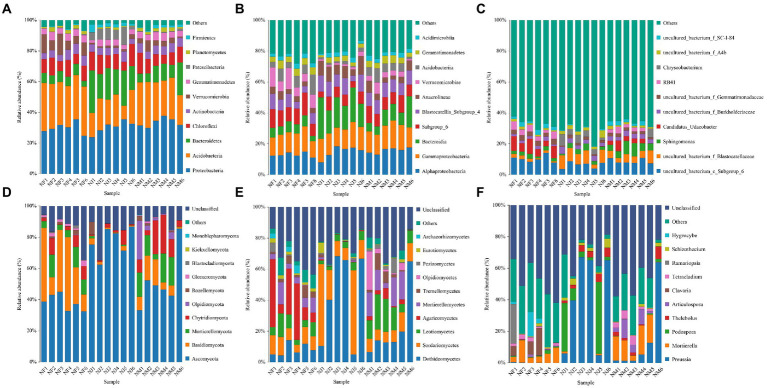
Histogram of the relative abundance distribution of species at different taxonomic levels. **(A–C)** Represented bacterial abundance at phylum, class, and genus levels, respectively. **(D–F)** Represented fungal abundance at phylum, class, and genus levels, respectively.

Mapping the top ten most abundant bacterial taxa onto the species distribution histogram at different taxonomical levels (phylum, class, and genus) revealed some clustering of community structure by excrement contamination (Figures 4A–C). Regardless of excrement contamination, Proteobacteria was the dominating phylum, accounting for more than 29.81 percent of all sequences. In addition, Acidobacteria was the most abundant phylum in NF (29.41%) and NM (25.29%), whereas NJ had the dominant phylum of Bacteroidetes (20.12%). The top 3 most abundant bacterial classes in NF were Alphaproteobacteria (12.96%), Gammaproteobacteria (13.22%), and Subgroup_6 (10.74%), while Alphaproteobacteria (15.83, 14.23%), Gammaproteobacteria (14.84, 14.25%), and Bacteroidia (10.26, 19.31%) dominated the NM and NJ. At the genus level, The top 5 most abundant bacterial genera identified in NF were *uncultured_bacterium_c_Subgroup_6* (10.16%), *Candidatus_Udaeobacter* (7.02%), *uncultured_bacterium_f_Gemmatimonadaceae* (3.06%), *RB41* (3.92%), and *uncultured_bacterium_f_SC-I-84* (2.32%); *uncultured_bacterium_c_Subgroup_6* (8.67%), *uncultured_bacterium_f_Blastocatellaceae* (5.18%), *Sphingomonas* (5.77%), *Candidatus_Udaeobacter* (3.01%), and *uncultured_bacterium_f_Burkholderiaceae* (2.92%) in NM, while *uncultured_bacterium_c_Subgroup_6* (6.25%), *uncultured_bacterium_f_Blastocatellaceae* (7.71%), *uncultured_bacterium_f_Burkholderiaceae* (3.11%), *Chryseobacterium* (4.35%), and *uncultured_bacterium_f_A4b* (2.66%) in NJ.

**Figure 5:**
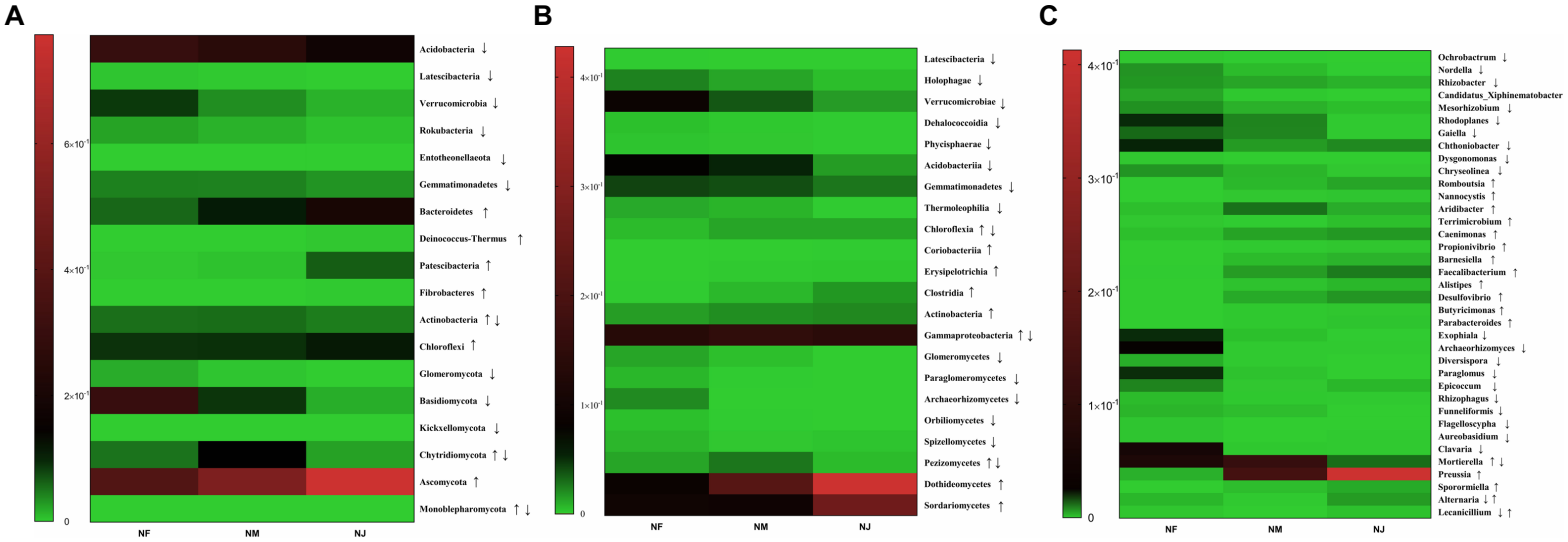
Heatmap of significant alterations in soil microorganisms (bacteria and fungi) at multiple classification levels as a function of excrement contamination. **(A)** Phylum level; **(B)** class level; **(C)** genus level. ↑/↓ represented that the abundance of taxa continued to significantly increase/decrease in NF, NM, NJ groups; ↑↓ represented that the abundance of taxa significantly increased firstly and decreased later in NF, NM, NJ groups; ↓↑ represented that the abundance of taxa significantly decreased firstly and increased later in NF, NM, NJ groups.

We used Metastst analysis to quantify the association of excrement contamination induced by intensive grazing with bacterial-microbiota composition. According to mapping results, the distribution of significantly altered clusters on the heatmap supported our hypothesis. At the phylum level, several taxonomic classifications in soil, Acidobacteria, Latescibacteria, Verrucomicrobia, Rokubacteria, Entotheonellaeota, and Gemmatimonadetes, significantly decreased with the increasing degree of excrement contamination (Figure 5A). In contrast, Bacteroidetes, Deinococcus-Thermus, Patescibacteria, Fibrobacteres, Actinobacteria, and Chloroflexi had the opposite trend (*p* < 0.05 or *p* < 0.01). Among the 14 classes affected by excretion contamination, the average relative abundance of 8 taxa (Figure 5B), Latescibacteria, Holophagae, Verrucomicrobiae, Dehalococcoidia, Phycisphaerae, Acidobacteriia, Gemmatimonadetes, and Thermoleophilia were lower in NM and NJ than in NF. At the same time, Chloroflexia, Coriobacteriia, Erysipelotrichia, Clostridia, Actinobacteria, and Gammaproteobacteria increased with the increasing degree of contamination (*p* < 0.05 or *p* < 0.01). Metastatic analysis confirmed the dominance of genera *Ochrobactrum*, *Nordella*, *Rhizobacter*, *Candidatus_Xiphinematobacter*, *Mesorhizobium*, *Rhodoplanes*, *Gaiella*, *Chthoniobacter*, *Dysgonomonas*, and *Chryseolinea* in the NF microbiota (Figure 5C). *Romboutsia*, *Nannocystis*, *Terrimicrobium*, *Caenimonas*, *Propionivibrio*, *Barnesiella*, *Faecalibacterium*, *Alistipes*, *Desulfovibrio*, *Butyricimonas*, and *Parabacteroides* were over-expressed (*p* < 0.05 or *p* < 0.01) among the 22 substantially shifted genus-level OTUs in NJ. NM and NJ could also be classified from each other, although the variations in relative abundance were not as pronounced as in the case NF versus NJ.

### Significant alterations in fungal-microbiota composition and dominant species under the influence of yaks’ excrement contamination

The phylum Ascomycota accounts for the overwhelming majority of the dominant soil microbiome in fungal communities, accounting for more than 38.19 percent of all stands. There were also three dominant phyla: Basidiomycota (NF: 30.25%, NM: 8.84%, NJ: 1.72%), Mortierellomycota (NF: 8.15%, NM: 11.44%, NJ: 0.46%), Chytridiomycota (NF: 5.18%, NM: 12.23%, NJ: 2.58%) within the fungal communities (Figure 4D). At the class level, Sordariomycetes (9.45%) and Agaricomycetes (21.86%) fungi were the most predominant in the NF, while Dothideomycetes (21.92%) and Leotiomycetes (13.91%) were found in the NM. Meanwhile, the class Dothideomycetes (43.18%) was the most abundant fungi in the NJ, followed by the Sordariomycetes (25.64%; Figure 4E). Following the genus-level classification results, the most abundant genera were *Mortierella* (6.65%), *Clavaria* (5.37%), *Ramariopsis* (4.35%), and *Hygrocybe* (3.41%) in the NF, *Preussia* (14.42%), *Mortierella* (11.36%), *Thelebolus* (2.38%), *Articulospora* (5.10%), and *Tetracladium* (2.96%) in the NM, while *Preussia* (41.74%), *Podospora* (14.96%), *Thelebolus* (3.75%), and *Schizothecium* (2.72%) were identified as the predominant in the NJ (Figure 4F).

The soil fungal-microbiota composition and dominant species assembly were then estimated for the three samples. According to the heatmap, the phyla Glomeromycota, Basidiomycota, and Kickxellomycota are the most abundant in NF soil microbiome (Figure 5A), Chytridiomycota, Monoblepharomycota were dominated in NM, while NJ had a large proportion of Ascomycota (*p* < 0.05 or *p* < 0.01). The class-level findings revealed a continuing enrichment in the relative abundances of the phyla Dothideomycetes and Sordariomycetes with increasing degrees of excrement contamination (Figure 5B), whereas Glomeromycetes, Paraglomeromycetes, Archaeorhizomycetes, Orbiliomycetes, Spizellomycetes, Pezizomycetes had the opposite trend (*p* < 0.05 or *p* < 0.01). *Exophiala*, *Archaeorhizomyces*, *Diversispora*, *Paraglomus*, *Epicoccum*, *Rhizophagus*, *Funneliformis*, *Flagelloscypha*, *Aureobasidium*, and *Clavaria* were the most prevalent genera in NF group. On the other hand, *Preussia*, *Sporormiella*, *Alternaria*, and *Lecanicillium* were relatively abundant in NJ group. Furthermore, *Mortierella* remained the predominant population in NM group (Figure 5C).

## Discussion

The plateau of Aba Autonomous Prefecture, China, only inhabits the traditional Tibetan people and a few Han Chinese due to the unique geographical and survival conditions. The abundant pasture resources allow these locals to herd grazing for their livelihood. Increasing evidence suggests that soil microbiome promotes ecosystem multi-functionality and regulates resistance to land-surface’s common disturbance practices. However, interference from fecal deposition excrement caused by extensive grazing may disrupt this context dependence throughout complex and harmoniously balanced ecosystems (Jing et al., 2015; Delgado-Baquerizo et al., 2016). The pressures associated with excrement contamination might deteriorate soil micro-ecology with time, affecting herbage yields substantially (Shah et al., 2017). So, the research into the mechanistic understanding of the connections between interfering ecological processes and variations in plateau soil microbial diversity is very important. This is the first in-depth study of bacterial and fungal taxonomic changes in excrement contamination soils in the plateau. According to our findings, excrement contamination significantly reduces soil bacterial and fungal diversity and richness, indicating that environmental filtering substantially impacts microbial community formation. We noticed significant differences in soil microbiome composition across groups, with NF soil communities separating the most from NM and NJ soil communities.

Grazing activities reduce soil microbial activity in three main ways: (1) soil compaction decreases aggregate stability index, nutrient availability, and porosity, which leads to the reduction of soil health (Shah et al., 2017), (2) it lowers plant performance *via* reduced root growth (Lai and Kumar, 2020), (3) excremental contamination (Wegrzyn et al., 2018). Based on this data, we hypothesized that excrement contamination, a kind of land-surface disturbance, might disrupt a complex and harmoniously balanced ecosystem in the plateau, affecting both the quality and diversity of below-ground microorganisms. Current data may support this hypothesis since NF group had significantly higher alpha diversity than both NM and NJ, with NJ being the worst of the three. Previous studies have shown that excrement contamination negatively impacts soil characteristics and microbial communities, suggesting strong soil microbiota response-specific patterns under the influence of land-surface disturbance practices (Dombos, 2001). Furthermore, the scatter plot represents the cluster of similarities and branches in the evolutionary tree, sequestering into three clusters with a higher degree of excrement contamination, indicating that this type of contamination drove the microbial composition and structural segregation in plateau soil.

Most soil microbiome studies have focused on a single counterpart (bacteria or fungi) without considering the multi-kingdom state of the soil ecosystem. Furthermore, many previous studies that have identified soil microbial variations focused on natural conditions such as stages of soil microecology development and plant community succession (Da et al., 2009; Rime et al., 2015). However, few studies have investigated soil microbial responses to land-surface disturbance practices. Here, we demonstrated clear alterations in bacteria and fungi communities driven by animal excrement contamination in the plateau. Identifying these processes aided us in understanding the soil microbial system resilience and contributed to the increasing evidence that soil microbial communities can change dramatically in response to soil ecological processes (Fierer et al., 2007).

As previously mentioned, the consensus is that the loss of functional microorganisms’ biodiversity residing below-ground reduces the functions of terrestrial ecosystems and destroys their long-term stability (Bardgett and van der Putten, 2014). This knowledge conveyed that the reduction in potential functional microbiota exacerbates the terrestrial ecological imbalance or that soil property harmed by land-surface disturbance practices causes a decrease in beneficial and functional microorganisms. The total of 12 bacterial phyla showed a significant change in relative abundance among three groups in our data, supported by previous work. The bacterial phyla Acidobacteria and Latescibacteria had previously been shown to be more abundant in grassland soils than in grazing soil, which the trend might associate with their functional roles in ecosystems [e.g., Acidobacteria had been proven to have a beneficial role in recovering soils to improve plant growth and nutrient cycling after surface disturbance (Huang et al., 2015); and Latescibacteria mediated the turnover of niche specialization in organic carbon that reaches deeper anoxic/microoxic habitats (Youssef et al., 2015)]. What data suggests differs from the multimetal resistance pathway in phylum Entotheonellaeota, phylum Verrucomicrobia including class Verrucomicrobiae, as metal-resistant bacteria could secrete EPS-proteins and EPS-polysaccharides to detoxify Cr, were associated with C/N cycling processes (Zhang et al., 2021). Heavy metals (including As, Cr, Cd, Hg, Cu, etc., in polluted soils) are ubiquity in various environmental mediums and attract attention in China and around the world for their toxicity, bioaccumulation, and carcinogenicity (Stefanowicz et al., 2008; Zhao et al., 2015). The interaction between microbiota and metal became important in contaminated field soils due to the various functions of soil microbes’ capacity to produce metal carbonate precipitation along with nitrogen cycling (Satapute et al., 2019). The significantly decreased abundance of microorganisms related to metal detoxification in our data might be attributed to the decreasing self-repairing ability of the soil ecosystem with the increasing degree of excrement contamination. So, the current conclusion is consistent with previously published results (Abdalla et al., 2018). Additionally, phylum Chloroflexi and class Chloroflexia were frequently dominant in methanogenic reactors, and they were also more abundant in NM and NJ than NF (Bovio et al., 2019). Similar to earlier studies, our findings revealed a negative response of soil pathogens to animal excrement contamination (Gagliardi and Karns, 2000). The decline in soil microbial diversity and beneficial microorganisms shifted dominance to soil pathogen-associated phylotypes, such as Coriobacteriia (Gardiner et al., 2015), Erysipelotrichia (Singh et al., 2020), Clostridia and Actinobacteria (Miao and Davies, 2010; Moore and Lacey, 2019). However, grazing was not the sole reason for increasing class-level pathogenic bacterium. Our data at the genus level further support animal excrement contamination that is further connected with significantly increased pathogens in the soil. Abundant *Desulfovibrio* is the inducer in intestinal pathogenesis, which can induce intestinal epithelial cells abnormal proliferation and metabolism and even impair intestinal barrier function (Gill et al., 2006). The relative abundance of *Romboutsia*, *Butyricimonas*, and *Parabacteroides* in soil significantly enriched with the increasing degree of excrement contamination, which might be dangerous information because these three genera pose a big threat to host health (Ricaboni et al., 2016; Kierzkowska et al., 2018; De Donder et al., 2020). More than 90% of the yaks in the world live in the plateaus of China (> 3,000 m), facing harsh environmental conditions, especially at calve stage (Wang et al., 2018). According to statistical research, diarrheal disease epidemic in calves in April (Li et al., 2018). The pathogenic bacteria in the host intestines can infiltrate the soil, even underground water bodies. As a result, excrement is a significant vector for disease transportation and has even become a serious public health issue (Lipp et al., 2001). Hence, we hypothesized a linkage between the significant abundance of soil pathogens and the susceptibility to intestinal diseases in calves’ yak. While suggesting that such soil pathogens impact the etiology of intestinal diseases in the host that needs further study.

The specimen collection in the current study avoided other surface disturbance activities, e.g., cultivation, man-made sabotage, ensuring that animal excrement was the main driver for altering the bacterial and fungal communities in this area. A common view is that fungi utilize the Carbon from the soil more effectively than other microbial groups. The main information to emerge is that the fungal community is highly sensitive to changes in environmental condition (Valentin-Vargas et al., 2014). Basidiomycota represents the dominant phylum of the fungal kingdom, which plays a significant role in medicinal properties, ecosystem functioning, and carbon recycling (Schmidt-Dannert, 2016). Ecologists began exploring soil fungi’s role in soil nutrient dynamics in the 1980s, revealing the function of microorganisms with an important role in stimulating nutrient mineralization and decomposition processes, e.g., Chytridiomycota and Kickxellomycota. The latter is positively associated with soil carbon, phosphorous, nitrogen, and potassium (Tarin et al., 2021). The significant decrease in the above-mentioned functional fungi in NM and NJ was of great concern since it conveyed that animal excrement contamination in the plateau gradually destroyed the soil ecosystem’s nutrient cycling.

**Figure 6:**
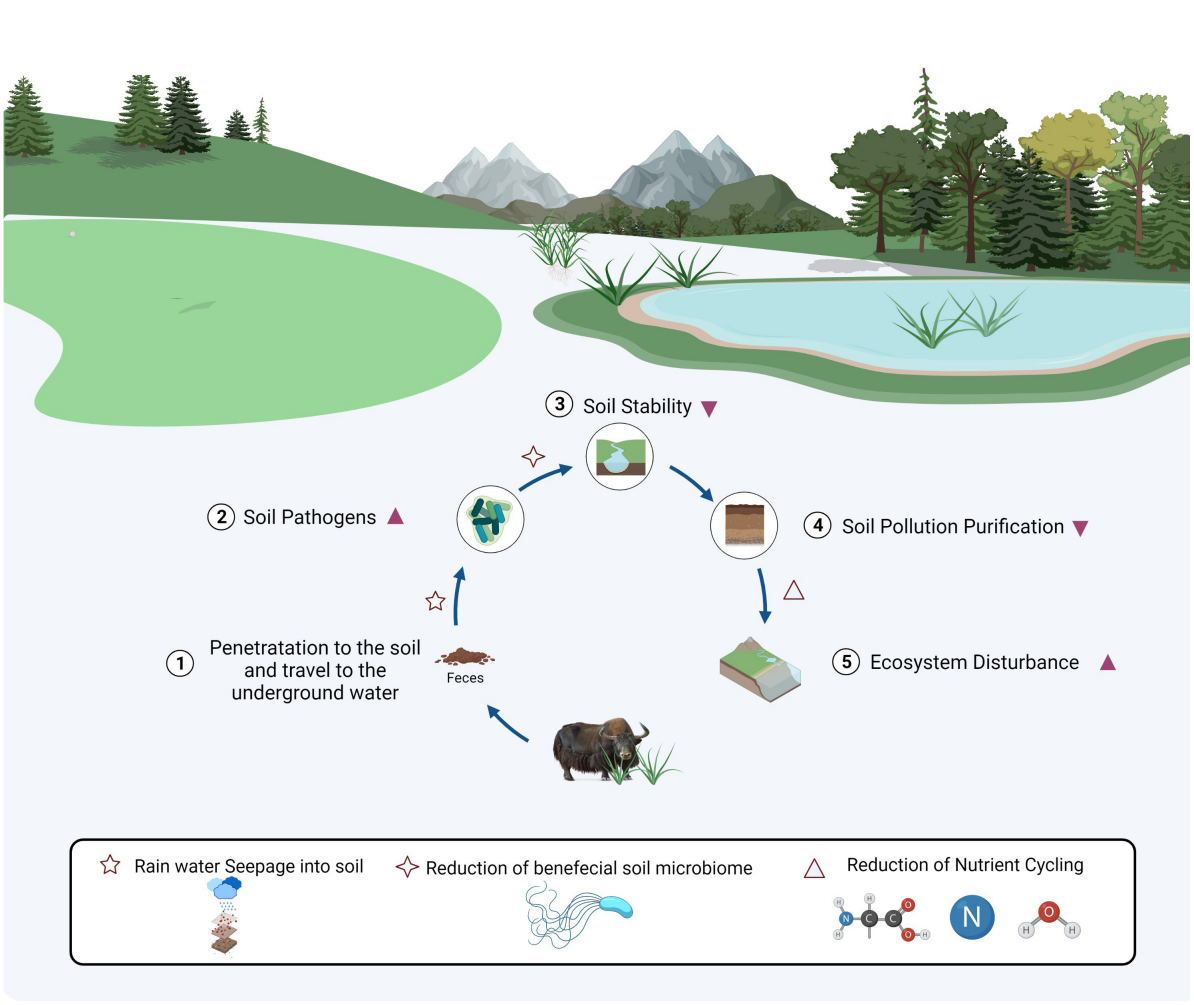
The mechanism insight of soil pathogens due to excrement contamination at plateau.

Furthermore, previous evidence demonstrated that intensive grazing could lower biomass and decomposition rate of plant roots, which reduce the soil Nitrogen (N) nutrients (NO3- and NH4-), subsequently reducing soil total N (He et al., 2012). Animal excretes increased N in the soil. However, the deposition rate is minor (Macdiarmid and Watkin, 1972; BALL et al., 1979). Therefore, the loss of nitrogen driven by grazing was more significant than nitrogen deposition in manure, ultimately leading to an overall reduction of soil N. Besides the impacts of fungi in soil nutrient dynamics, several beneficial microorganisms, i.e., phylum Glomeromycota along with classes Glomeromycetes and Paraglomeromycetes, and genera *Diversispora* and *Paraglomus* could be used in rhizostabilisation strategies to reduce soil waste heaps and extreme metal contamination, which pollution is a permanent threat for nearby surroundings (Sanchez-Castro et al., 2017). The genus *Epicoccum* has produced multi metabolites with potential biotechnological applications, e.g., anticancer, antioxidant, and antimicrobial compounds. It is mainly known as biocontrol agent against phytopathogens (Braga et al., 2018). Arbuscular mycorrhizal fungi, obligatory plant symbionts, e.g., *Rhizophagus* and *Funneliformis*, have a beneficial impact on plants in contaminated conditions (Malicka et al., 2020); while bacterial genera *Rhodoplanes* and *Gaiella* are predominant in the rhizospheric soils, accelerates the degradation of herbicide (atrazine) residues (Aguiar et al., 2020). The relationships of pathogens-pollutants-microbes might be described as a disease triangle, and soil pollution indirectly or directly interacts with pathogens in soil through containing impacts on the host plant (Dean et al., 2012). It was also demonstrated that amount of soil-borne pathogens differed significantly between NM and NJ groups, with the highest abundance in NJ group, i.e., phylum Monoblepharomycota classes Dothideomycetes and Sordariomycetes, and genera *Alternaria* and *Lecanicillium*, among which vegetable foods infected by *Alternaria* conveyed host-specific toxins to the human diet (Van Rooij et al., 2015; Weber et al., 2015; Pinto and Patriarca, 2017; Haridas et al., 2020; Zhou et al., 2020). When emphasizing on soil community variability, the opposite had to include soil system resilience. It is a resistance/tolerance of soil immune responses against biotic infections such as microbiome-mediated degradation of pollutants (Raaijmakers and Mazzola, 2016). Soil pollution, as an invasive element wreaking on the soil ecology, may be mitigated by the soil microbiome (Wang and Li, 2019). However, in the current study, the strong resilience of the soil microbial community did not conceal the detrimental impacts of surface disturbance, suggesting that the destructive effect of excremental contamination exceeds resilience in plateau soil (Figure 6). With many selection pressures imposed by animal excrement contamination and soil-borne diseases, we predicted that a limited soil ecological niche with more pathogens and a lower functioning microbiome would emerge (Wood et al., 2016).

## Conclusion

Our study first demonstrated the dynamic dispersion of soil microbiomes, which comprised bacterial and fungal communities in soils disturbed by land-surface practices (excrement contamination induced by grazing) on the plateau. Specifically, our findings that excrement contamination reduced soil diversity and richness of bacterial and fungal communities indicated that land-surface disturbance practices had a major impact on microbial community formation. Furthermore, a continuous decrease in the relative abundances of microorganisms associated with ecological nutrient cycling, soil pollution purification, and root soil stability continue to decline with the increasing degree of excrement contamination, whereas soil pathogens had the opposite trend in grazing. The alterations in the dominant species and composition of the soil microbiome supported the concept that animal excrement contamination induced a low diversity and richness with more pathogens and lower functioning microbiome in the soil microbiome. Such colonization and succession of the soil excrement contamination induce microbiome may offer an important potential management basis for microbiome-based soil health in the plateau. However, the limitation of current research is the lack of exploration and validation of the effects of excrement contamination on soil functionality in the plateau, and further experiments are still needed.

## Data availability statement

The datasets presented in this study can be found in online repositories. The names of the repository/repositories and accession number(s) can be found at: https://www.ncbi.nlm.nih.gov/, PRJNA772904.

## Author contributions

ZS, YW, MA, KL, and SL provided the research idea. ZT and XC contributed reagents, materials, and analysis tools. YW wrote the manuscript. MK revised the manuscript. All authors contributed to the article and approved the submitted version.

## Funding

This study was supported by Start-up fund of Nanjing Agricultural University (804131), Start-up Fund for Distinguished Scholars of Nanjing Agricultural University (80900219), Tibet Regional Science and Technology Collaborative Innovation Project (QYXTZX-NQ2021-01) and The Central Government supports the special fund project for the reform and development of local universities (KY2022ZY-01).

## Conflict of interest

The authors declare that the research was conducted in the absence of any commercial or financial relationships that could be construed as a potential conflict of interest.

## Publisher’s note

All claims expressed in this article are solely those of the authors and do not necessarily represent those of their affiliated organizations, or those of the publisher, the editors and the reviewers. Any product that may be evaluated in this article, or claim that may be made by its manufacturer, is not guaranteed or endorsed by the publisher.

## Supplementary material

The Supplementary material for this article can be found online at: https://www.frontiersin.org/articles/10.3389/fmicb.2022.1016852/full#supplementary-material
