# Supplementary material for: The successional trajectory of bacterial and fungal communities in soil are fabricated by yaks’ excrement contamination in plateau, China
Source: Front Microbiol. 2022 Nov 17;13:1016852. doi: 10.3389/fmicb.2022.1016852 (PMC9714268; doi:10.3389/fmicb.2022.1016852)
Supplement: Supplementary file 1 [file Data_Sheet_1.doc]

Supplementary table 1. The information of bacterial sequencing data.

| Sample ID | Raw Reads | Clean Reads | Effective Reads | AvgLen(bp) | Effective(%) |
| --- | --- | --- | --- | --- | --- |
| NF1 | 80,268 | 80,062 | 78,605 | 421 | 97.93 |
| NF2 | 79,609 | 79,345 | 75,832 | 419 | 95.26 |
| NF3 | 79,861 | 79,656 | 77,437 | 421 | 96.96 |
| NF4 | 80,451 | 80,193 | 76,919 | 420 | 95.61 |
| NF5 | 80,118 | 79,884 | 75,821 | 420 | 94.64 |
| NF6 | 79,574 | 79,345 | 76,393 | 419 | 96 |
| NJ1 | 79,866 | 79,643 | 77,538 | 416 | 97.09 |
| NJ2 | 79,948 | 79,711 | 73,133 | 417 | 91.48 |
| NJ3 | 73,657 | 73,443 | 65,382 | 417 | 88.77 |
| NJ4 | 79,994 | 79,746 | 70,920 | 417 | 88.66 |
| NJ5 | 55,122 | 54,935 | 48,307 | 417 | 87.64 |
| NJ6 | 80,118 | 79,887 | 76,108 | 416 | 94.99 |
| NM1 | 80,175 | 79,912 | 75,750 | 418 | 94.48 |
| NM2 | 80,091 | 79,856 | 76,200 | 419 | 95.14 |
| NM3 | 80,080 | 79,820 | 73,972 | 419 | 92.37 |
| NM4 | 79,969 | 79,719 | 72,590 | 419 | 90.77 |
| NM5 | 80,005 | 79,754 | 75,722 | 419 | 94.65 |
| NM6 | 79,834 | 79,614 | 71,447 | 417 | 89.49 |

Supplementary table 2. The information of fungal sequencing data.

| Sample ID | Raw Reads | Clean Reads | Effective Reads | AvgLen(bp) | Effective(%) |
| --- | --- | --- | --- | --- | --- |
| NF1 | 80,032 | 79,754 | 79,225 | 245 | 98.99 |
| NF2 | 79,846 | 79,560 | 77,376 | 264 | 96.91 |
| NF3 | 80,168 | 79,892 | 79,449 | 256 | 99.1 |
| NF4 | 79,820 | 79,540 | 78,927 | 259 | 98.88 |
| NF5 | 80,231 | 79,957 | 78,706 | 256 | 98.1 |
| NF6 | 79,816 | 79,513 | 76,593 | 253 | 95.96 |
| NJ1 | 80,317 | 80,020 | 79,204 | 244 | 98.61 |
| NJ2 | 79,817 | 79,545 | 78,784 | 244 | 98.71 |
| NJ4 | 80,031 | 79,807 | 79,276 | 226 | 99.06 |
| NJ5 | 79,874 | 79,537 | 78,799 | 244 | 98.65 |
| NJ6 | 79,938 | 79,687 | 78,917 | 225 | 98.72 |
| NM1 | 80,032 | 79,765 | 78,362 | 239 | 97.91 |
| NM2 | 80,141 | 79,849 | 76,794 | 251 | 95.82 |
| NM3 | 79,562 | 79,292 | 78,421 | 254 | 98.57 |
| NM4 | 79,933 | 79,663 | 77,610 | 260 | 97.09 |
| NM5 | 79,904 | 79,567 | 78,114 | 253 | 97.76 |
| NM6 | 79,970 | 79,672 | 78,579 | 236 | 98.26 |


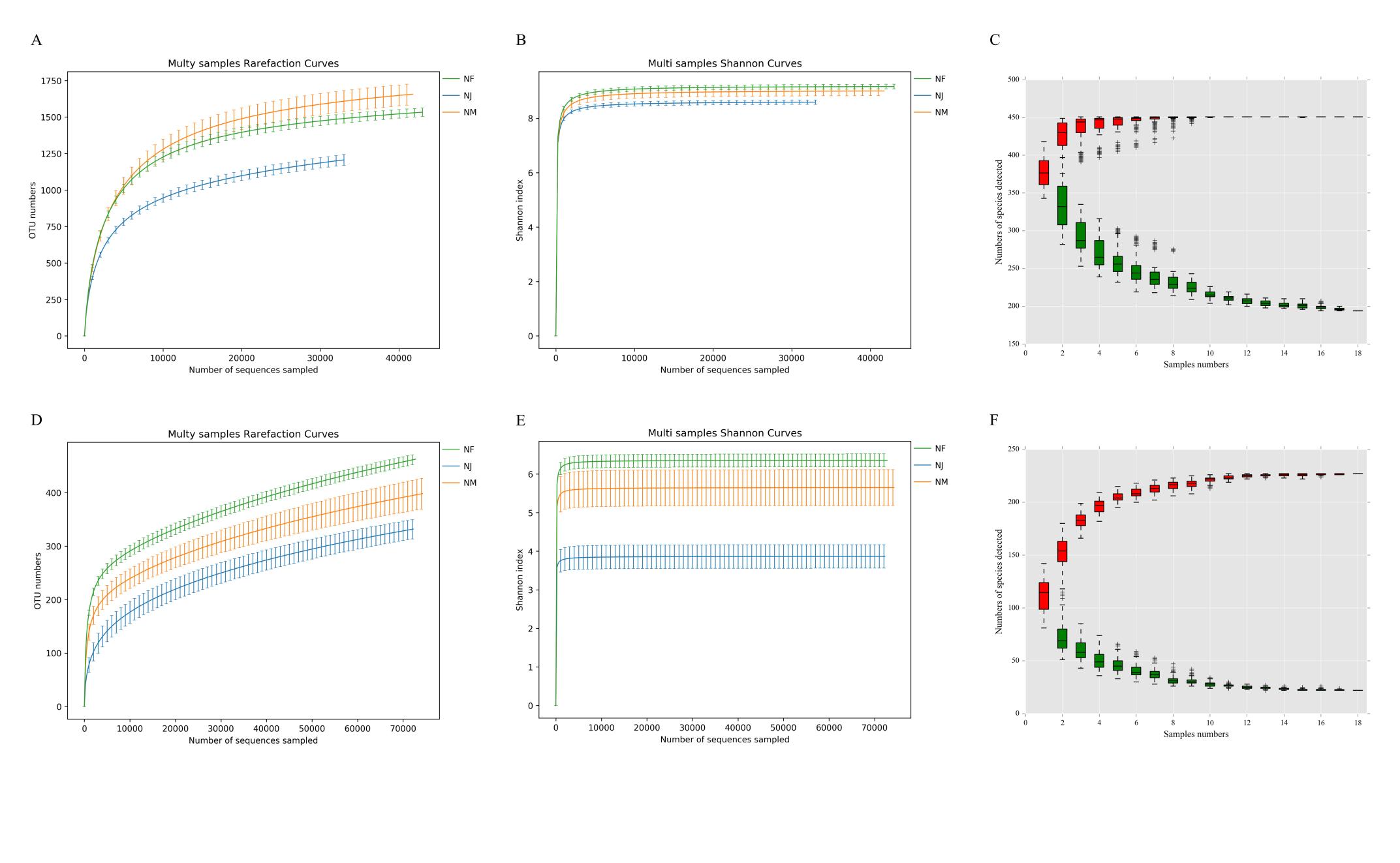


Supplementary Fig. 1 Sample feasibility analysis. A, B, C represented the bacterial Rarefaction curve, Shannon index, and Rank abundance curve. D, E, F represented the fungal rarefaction curve, Shannon index, and rank abundance curve.


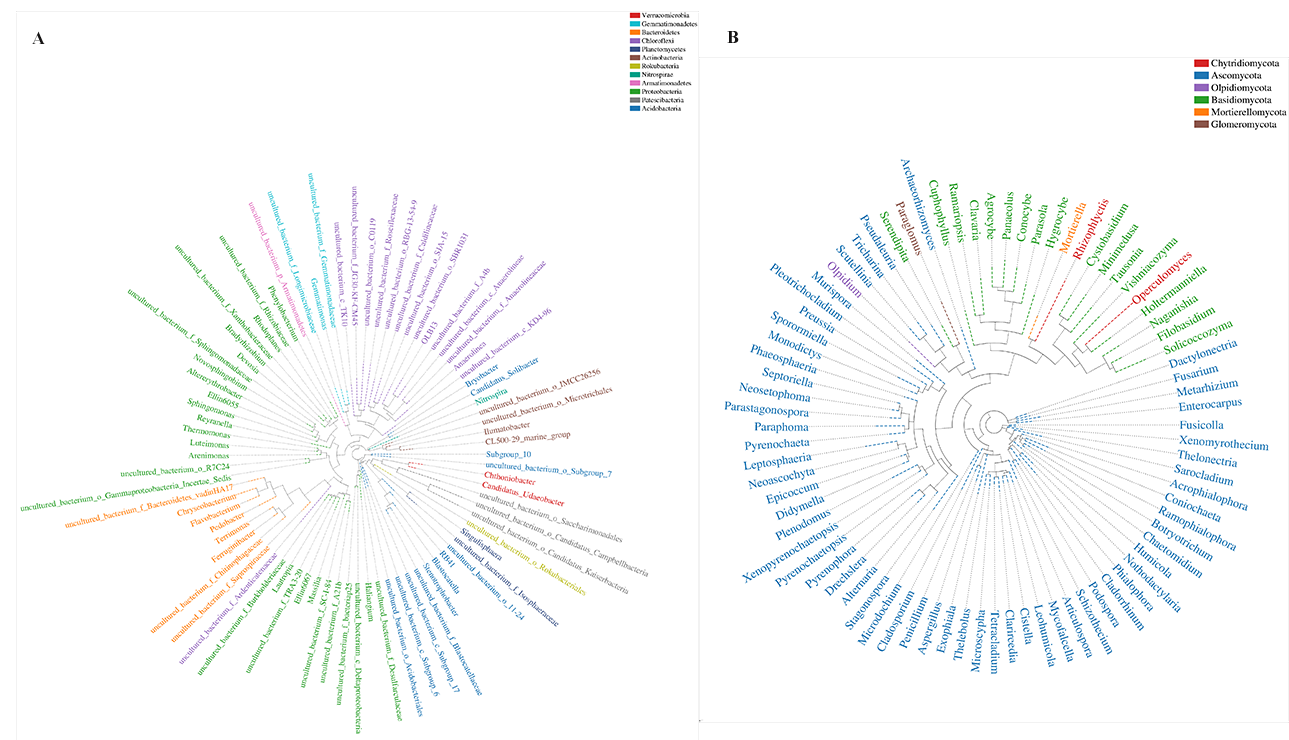


Supplementary Fig. 2 genus-level phylogenetic tree. (A) represented bacterial genus-level phylogenetic tree; (B) represented fungal genus-level phylogenetic tree.
